# Accuracy of cephalometric landmark identification on artificial intelligence-based software: a comparative study

**DOI:** 10.1590/2177-6709.31.2.e2625144.oar

**Published:** 2026-05-18

**Authors:** İpek ŞAVKAN, Sara Nur ÖZÇANKAYA, Öykü Naz TURAN

**Affiliations:** 1 İstanbul Kent University, Faculty of Dentistry, Department of Orthodontics (Istanbul, Türkiye).; 2 Istanbul University, Faculty of Dentistry, Department of Orthodontics (Istanbul, Türkiye).

**Keywords:** Cephalometrics, Orthodontic imaging, Artificial intelligence, Diagnostic records, Digitalization, Cefalometria, Imaginologia ortodôntica, Inteligência artificial, Registros diagnósticos, Digitalização

## Abstract

**Introduction:**

Cephalometry has long been a cornerstone of orthodontic diagnosis and treatment planning. While traditionally performed manually, cephalometric analysis has increasingly shifted toward semi-automated or fully automated computer-based software, due to advances in technology.

**Objective:**

This study aims to evaluate and compare the accuracy of anatomical landmarks identified manually by orthodontists using digital tools versus those identified automatically by various artificial intelligence (AI)-based cephalometric software.

**Methods:**

A total of 25 lateral cephalometric radiographs were selected for analysis. Manual landmark identification was independently performed by two orthodontists using the digital cephalometric software NemoCeph. These were compared to the automatic landmark identifications generated by two AI-based programs: CephNinja and WebCeph™. A total of 17 anatomical landmarks, 15 hard tissue and 2 soft tissue points, were evaluated. The 17 cephalometric landmarks selected in this study are among the most commonly used and reliably identifiable reference points in the literature. To facilitate direct visual comparison, landmark positions derived from all three methods were overlaid using semi-transparent image layers. One-way ANOVA and intraclass correlation coefficient (ICC) tests were conducted to assess inter-method differences. The threshold for statistical significance was set at p < 0.05 and p < 0.01.

**Results:**

Statistically significant differences were observed at the porion, basion, gonion, and the apexes of the upper and lower incisors (p < 0.05). Compared to the manual tracings in NemoCeph, AI-based software generally placed these landmarks in more distal and superior positions. Among the AI tools, CephNinja showed greater similarity to NemoCeph for the gonion and basion points, while WebCeph™ was closer at the incisor apexes.

**Conclusion:**

Although AI-based cephalometric software offers rapid and reproducible assessments, certain anatomical landmarks - particularly porion, basion, gonion, and the apexes of the upper and lower incisors - demonstrate statistically significant positional discrepancies, compared to manual tracings. Clinicians should be cautious when relying exclusively on automated analyses for diagnostic or treatment planning purposes, since AI struggles with certain landmarks, due to algorithmic limitations.

## RESUMO

**Introdução:**

A cefalometria tem sido, por muito tempo, um pilar do diagnóstico e do planejamento do tratamento ortodôntico. Embora tradicionalmente realizada de forma manual, a análise cefalométrica tem migrado cada vez mais para *softwares* computadorizados semiautomatizados ou totalmente automatizados, devido aos avanços tecnológicos.

**Objetivo:**

Este estudo teve como objetivo avaliar e comparar a acurácia dos marcos anatômicos identificados manualmente por ortodontistas por meio de ferramentas digitais *versus* aqueles identificados automaticamente por diversos *softwares* cefalométricos baseados em inteligência artificial (IA).

**Métodos:**

Um total de 25 telerradiografias laterais foram selecionadas para análise. A identificação manual dos marcos anatômicos foi realizada de forma independente por dois ortodontistas, utilizando o *software* de cefalometria digital NemoCeph. Essas foram comparadas às identificações automáticas de marcos geradas por dois programas baseados em IA: CephNinja e WebCeph™. Foram avaliados 17 marcos anatômicos no total, sendo 15 em tecido duro e 2 em tecido mole. Os 17 marcos cefalométricos selecionados neste estudo estão entre os pontos de referência mais comumente utilizados e citados na literatura. Para facilitar a comparação visual direta, as posições dos marcos derivadas dos três métodos foram sobrepostas utilizando camadas de imagem semitransparentes. Os testes ANOVA de uma via e o coeficiente de correlação intraclasse (ICC) foram utilizados para avaliar as diferenças entre os métodos. O nível de significância estatística foi definido em p < 0,05 e p < 0,01.

**Resultados:**

Diferenças estatisticamente significativas foram observadas no pório, básio, gônio e nos ápices dos incisivos superiores e inferiores (p < 0,05). Em comparação aos traçados manuais no NemoCeph, os *softwares* baseados em IA geralmente posicionaram esses marcos em localizações mais distais e superiores. Entre as ferramentas de IA, o CephNinja apresentou maior similaridade com o NemoCeph para os pontos gônio e básio, enquanto o WebCeph™ foi mais próximo nos ápices dos incisivos.

**Conclusões:**

Embora os *softwares* cefalométricos baseados em IA ofereçam avaliações rápidas e reprodutíveis, certos marcos anatômicos - particularmente o pório, o básio, o gônio e os ápices dos incisivos superiores e inferiores - demonstram discrepâncias posicionais estatisticamente significativas, em comparação aos traçados manuais. Os clínicos devem ser cautelosos ao confiar exclusivamente em análises automatizadas para fins de diagnóstico ou planejamento de tratamento, uma vez que a IA apresenta dificuldades com certos marcos, devido a limitações algorítmicas.

## INTRODUCTION

Although radiography was initially designed as a research laboratory tool, it has now become an indispensable diagnostic method in orthodontics, particularly for treatment planning and growth assessment.^1^ Lateral cephalometric radiography, which captures a side-view image of the skull, is a vital imaging technique used to evaluate sagittal and vertical craniofacial relationships. Cephalometry has long been a central tool for assessing dental and skeletal development, post-treatment changes, and for supporting clinical and research studies.^2^

Traditional cephalometric analysis begins with the manual identification of anatomical landmarks on specialized tracing paper, followed by angular and linear measurements using a ruler and compass. Although this method is still considered the gold standard in many studies, it is time-consuming and associated with a higher risk of error in landmark identification.^3^

With the advent of digital technology, semi-automated and fully automated cephalometric analysis tools have increasingly replaced manual tracing methods.^4^ Software such as NemoCeph, although not artificial intelligence (AI)-based, allows for digital manual landmark identification and measurement, offering increased efficiency and consistency. These tools reduce manual effort while still requiring user input and validation, thus ensuring accuracy through a combination of automation and human oversight.

Today, AI has emerged as a powerful tool across many domains, including Orthodontics. AI is defined as the science and engineering of creating intelligent machines, particularly smart computer programs.^5^ In Orthodontics, one of the most prominent applications of AI is automated cephalometric analysis.

Several commercially available AI-based software programs - such as CephX^®^ (ORCA Dental AI, Las Vegas, USA), AudaxCeph (Audax, Ljubljana, Slovenia), WebCeph™ (Assemblecircle, Seoul, Korea), and CephNinja (Cyncronus LLC, United States) - offer automatic reference point detection.^6^ These AI systems are designed to automatically identify cephalometric landmarks, lines, and angles on radiographs, reducing human error and saving time.

Moreover, AI models can be adapted by clinicians to suit specific patient characteristics or clinical conditions, enabling the development of more individualized and precise treatment plans.^7^

Unlike previous studies that focused primarily on angular or linear measurements, the present study uniquely evaluated positional discrepancies using a coordinate-based approach across multiple commercial AI platforms, offering a more precise understanding of landmarking inconsistencies.^8^

This study aimed to evaluate and compare the accuracy of anatomical landmark identification performed manually by experienced orthodontists and automatically by different AI-based cephalometric software programs.

## MATERIAL AND METHODS

 Ethical approval was obtained from the Istanbul Kent University (Approval No: 2024-06)

This study was based on a comparison between manual tracings performed by two orthodontists and automatic tracings produced by various AI-based cephalometric software programs. Twenty-five lateral cephalometric radiographs were selected using a simple random sampling method from an anonymized archive of radiographs taken at Kent University’s Department of Orthodontics. The radiographs were obtained over a six-month period, between June 2024 and December 2024. Inclusion criteria were: high image quality, clear visibility of anatomical landmarks, and no history of craniofacial anomalies or prior orthodontic treatment.

Exclusion criteria included radiographs with poor image quality, patients with a history of surgical intervention, pregnancy, radiation sensitivity, or craniofacial syndromes. No restrictions were placed regarding age or gender. These criteria were designed to ensure sample homogeneity and to improve data reliability.

The required sample size was calculated using G*Power software. *A priori* power analysis indicated that a minimum of 25 radiographs would be sufficient to detect large effect sizes (effect size = 0.95) with a power of 95% at an alpha level of 0.05. This calculation assumed moderate variability in landmark positioning across methods.

**Figure 1:**
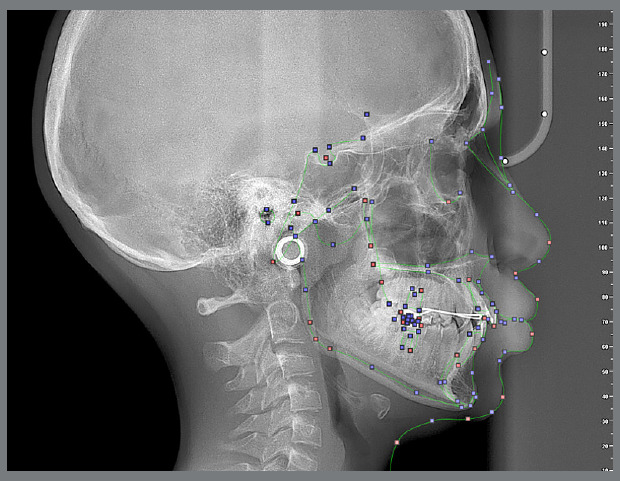
Anatomical reference points traced using the Nemoceph software.

Two orthodontists, each with a minimum of 5 years of clinical experience, independently traced the same radiographs using the NemoCeph software (Nemotec, Madrid, Spain) (Fig. 1). Each tracing was repeated after a 15-day interval, and the intra- and inter-observer reliability was assessed using the Intraclass Correlation Coefficient (ICC) (Table 1).

**Table 1:** Intraclass Correlation Coefficient ( ICC ).

	ICC	p
Sella	0.846	0.001**
Nasion	0.823	0.001**
Orbita	0.888	0.001**
Basion	0.947	0.001**
Porion	0.915	0.001**
Gonion	0.904	0.001**
Menton	0.875	0.001**
Gnathion	0.867	0.001**
Pogonion	0.840	0.001**
A Point	0.861	0.001**
B Point	0.836	0.001**
Tip of upper incisor	0.874	0.001**
Apex of upper incisor	0.841	0.001**
Tip of lower incisor	0.898	0.001**
Apex of lower incisor	0.941	0.001**
Subnasale	0.908	0.001**
Nasal tip	0.887	0.001**

**Figure 2:**
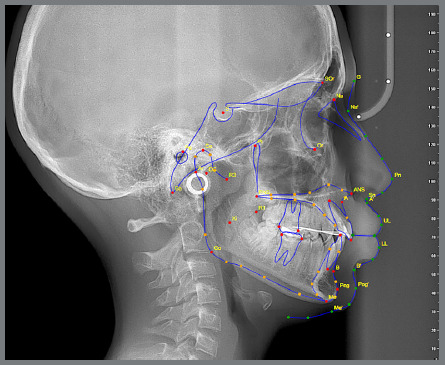
Anatomical reference points traced using the Webceph software.

**Figure 3:**
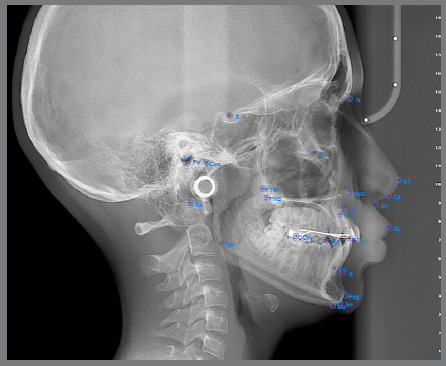
Anatomical reference points traced using the CephNinja software.

The same films were then analyzed using two AI-based cephalometric programs: WebCeph™ (Assemblecircle, Seoul, Korea) (Fig. 2) and CephNinja (Cyncronus LLC, USA) (Fig. 3). Seventeen cephalometric landmarks selected in this study are among the most commonly used and reliably identifiable reference points in the literature (Table 2). In the AI software, anatomical landmarks were automatically placed and not altered by the researchers. However, in the manual tracings, orthodontists were allowed to adjust landmark positions to reflect the most anatomically accurate placement.

**Table 2:** Anatomical landmarks.

Landmark	Description
Sella	Center of the sella turcica
Nasion	Intersection of the frontal and nasal bones
Porion	Upper margin of the external auditory meatus
Basion	Most inferior-posterior point of the foramen magnum
Gonion	Most posterior-inferior point on the angle of the mandible
Gnathion	Midpoint between the most anterior and inferior points of the chin
Menton	Most inferior point on the mandibular symphysis
Pogonion	Most anterior point on the chin
A Point	Deepest point on the curvature of the anterior maxilla
B Point	Deepest point on the curvature of the anterior mandible
Orbita	Lowest point on the infraorbital rim
Tip of upper incisor	Incisal edge of the most prominent upper central incisor
Apex of upper incisor	Root apex of the most prominent upper central incisor
Tip of lower incisor	Incisal edge of the most prominent lower central incisor
Apex of lower incisor	Root apex of the most prominent lower central incisor
Subnasale	Junction of the columella base and the upper lip
Nasal tip	Most anterior point of the nose

**Figure 4:**
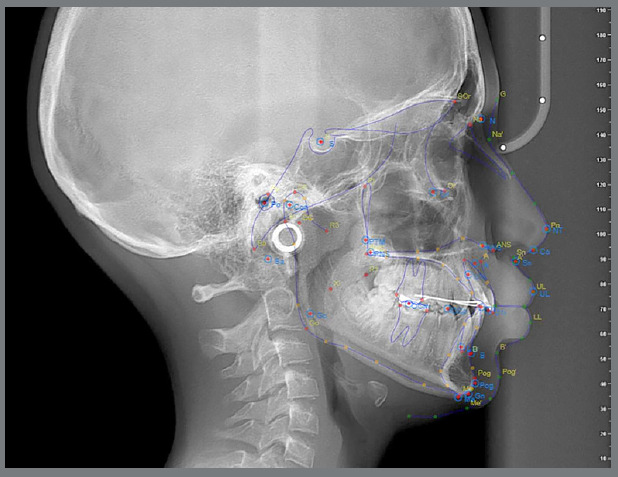
Overlay of analyses performed using the Nemoceph, Cephninja, and Webceph software programs.

The traced radiographs were overlaid across the three software platforms to visually compare the positioning of each landmark. The images were made semi-transparent, and alignment was verified using the reference rulers embedded in each software (Fig. 4).

The X and Y coordinates of each anatomical landmark were recorded separately for each software program. The distances were measured in millimeters. The positional differences (error margins) between the coordinates of the same landmark across different programs were calculated and compared.

## STATISTICAL ANALYSIS

All statistical analyses were conducted using SPSS version 24.0 (IBM Corp., Armonk, NY, USA). Descriptive statistics including mean, standard deviation, median, frequency, ratio, minimum, and maximum were computed.

To compare the coordinates of landmarks across the three software programs, one-way ANOVA was applied, followed by Bonferroni correction for *post-hoc* pairwise comparisons.

To assess intra- and inter-observer reliability, Intraclass Correlation Coefficient (ICC) values were calculated.

Statistical significance was evaluated at p < 0.05 and p < 0.01 levels.

## RESULTS

**Figure 5:**
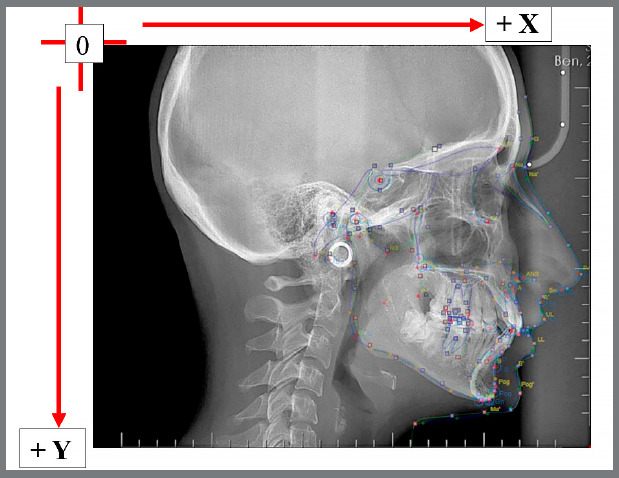
Superimposition of lateral cephalometric films obtained with Cephninja, WebCeph, and Nemo programs, and the use of a coordinate system.

According to the coordinate system used in this study, X-axis values increased from posterior to anterior, while Y-axis values increased from cranial to caudal (Fig. 5).

Statistically significant differences were found in the anatomical positions of the following landmarks: porion, basion, gonion, apex of the upper incisor, and apex of the lower incisor (p < 0.05) (Table 3).

**Table 3:** Comparison of anatomical landmark coordinates.

Coordinate values of anatomical points					
		NemoCeph	CephNinja	WebCeph	^a^ **p**
		Mean ± SD	Mean ± SD	Mean ± SD	
Sella	X-axis	1162.36±332.07	1162.16±332.28	1161.96±332.04	0.920
	Y-axis	410.8±83.53	410.32±82.08	410.88±83.13	0.932
Nasion	X-axis	1353.96±384.84	1354.84±384.27	1349.48±382.94	0.945
	Y-axis	381.16±82.33	379.84±79.61	381.28±79.7	0.956
Porion	X-axis	1101±312.46	1012.04±313.69	1050.88±313.37	0.044*
	Y-axis	465.32±95.43	435.6±99.51	426.72±94.97	0.032*
Basion	X-axis	1098.84±314.71	1058.36±314.56	1055.44±309.89	0.034*
	Y-axis	525.8±109.78	507.72±113.14	510.08±112.05	0.037*
Gonion	X-axis	1155.56±323.09	1131.44±326.67	1125.32±323.1	0.035*
	Y-axis	623.32±134.97	611.6±137.95	608.44±137.2	0.039*
Gnathion	X-axis	1335.48±379.35	1335.44±379.71	1335.4±378.27	0.975
	Y-axis	702.96±155.94	702.76±156.27	702.08±157.43	0.912
Menton	X-axis	1324.88±375.08	1323.44±375.63	1324.92±374.55	0.981
	Y-axis	705.52±158.07	705.24±157.98	704.88±156.93	0.989
Pogonion	X-axis	1342.64±381.03	1343.24±382.93	1341.72±380.95	0.991
	Y-axis	689.68±153.26	690.44±152.93	685.44±151.42	0.992
A Point	X-axis	1382.92±408.72	1384±407.2	1387.24±408.72	0.994
	Y-axis	552.16±124.16	551.72±122.53	553.16±122.57	0.931
B Point	X-axis	1334.64±383.37	1336.24±383.83	1334.8±382.84	0.978
	Y-axis	655.64±143.5	654±144.11	652.24±143.47	0.922
Orbita	X-axis	1310.6±375.47	1298.56±371.65	1313±373.09	0.989
	Y-axis	460.88±95.15	466.76±97.91	463.24±96.7	0.977
Tip of upper incisor	X-axis	1362.96±391.25	1361.96±391.32	1362.56±391	0.978
	Y-axis	607.24±132.72	604.4±132.26	606.56±132.85	0.992
Apex of upper incisor	X-axis	1336.52±382.02	1312.68±384.51	1327.12±382.61	0.041*
	Y-axis	542.96±115.21	527.96±121.61	536.24±116.72	0.039*
Tip of lower incisor	X-axis	1350.44±389.04	1350.88±389.53	1349.76±388.17	0.932
	Y-axis	599.4±130.63	602.12±130.74	599.64±129.83	0.997
Apex of lower incisor	X-axis	1319.24±377.26	1300.64±378.88	1305.48±379.92	0.039*
	Y-axis	657.24±143.62	639.76±141.16	647.36±142.8	0.045*
Subnasale	X-axis	1399±395.35	1396.44±396.64	1399.84±396.17	0.939
	Y-axis	542±116.07	550.84±116.08	544.12±119.77	0.962
Nasal tip	X-axis	1434.36±396.88	1433.84±398.43	1435.24±398.55	0.957
	Y-axis	512.12±107.32	510.44±106.87	510.2±106.04	0.998

^a^ One-way ANOVA test.

For the porion point, the mean coordinates on both X and Y axes differed significantly across the three software programs (p = 0.044 for the X-axis; p = 0.032 for the Y-axis). According to pairwise comparisons, AI-based software localized this point more distally and superiorly than the manual software. The coordinates determined by CephNinja were closer to those of NemoCeph in both the X and Y directions.

For the basion point, statistically significant differences in both X and Y coordinates were also observed (p = 0.034 for the X-axis; p = 0.037 for the Y-axis). AI-based programs again positioned this landmark more distally and superiorly, compared to manual tracings. However, in X direction, CephNinja showed greater similarity to NemoCeph, while in the Y direction, WebCeph™ was more similar to NemoCeph.

Similarly, the gonion point was identified more distally and superiorly by both AI-based software programs. CephNinja’s coordinate values in both X and Y directions were closer to those of NemoCeph (p = 0.035 for the X-axis; p = 0.039 for the Y-axis).

The apex of the upper incisor was also located in a more distal and superior position by both AI software programs. Among them, WebCeph™ provided closer coordinate values to NemoCeph in both axes (p = 0.041 for the X-axis; p = 0.039 for the Y-axis).

For the apex of the lower incisor, significant differences were again observed in both axes (p = 0.039 for the X-axis; p = 0.045 for the Y-axis). According to the pairwise comparison, this point was also more distally and superiorly positioned by the AI software, with WebCeph™ providing closer values to NemoCeph than CephNinja.

Intraclass correlation coefficient (ICC) values for all reference points were found to be close to 1, indicating high intra- and inter-observer agreement (p < 0.01).

*Post-hoc* analyses comparing software outputs for each anatomical landmark are presented in Table 4.

**Table 4:** Post Hoc Analysis Coordinate Values of Anatomical Points.

		NemoCeph - CephNinja	NemoCeph - WebCeph	CephNinja - WebCeph
Porion	X-axis	0.039	0.041	p>0.05
	Y-axis	0.021	0.035	p>0.05
Basion	X-axis	0.031	0.037	p>0.05
	Y-axis	0.027	0.033	p>0.05
Gonion	X-axis	0.031	0.044	p>0.05
	Y-axis	0.043	0.049	p>0.05
Apex of upper incisor	X-axis	0.035	0.046	p>0.05
	Y-axis	0.027	0.039	p>0.05
Apex of lower incisor	X-axis	0.039	0.041	p>0.05
	Y-axis	0.022	0.037	p>0.05

## DISCUSSION

Lateral cephalometric radiographs continue to play a key role in orthodontic diagnosis and treatment planning. However, the reliability of these analyses depends heavily on correctly identifying anatomical landmarks. Several studies have pointed out the limitations of conventional methods, especially when it comes to the consistency of landmark placement and measurement accuracy.^9^ ^,^ ^10^

Fortunately, with advances in technology, digital systems have made landmark identification and measurements faster and often more reliable.^11^ ^,^ ^12^ Many digital software programs have been developed, and a large body of research has confirmed that these tools can be as dependable as traditional manual methods. For example, software such as FACAD^®^, Cef-X 2001, and PACS have all demonstrated consistent results, when compared to manual tracing, making them suitable for clinical use.^13^ ^-^ ^16^

Other digital programs like Dolphin Imaging^®^, NemoCeph^®^, and AutoCEPH^®^ have also been widely tested for their performance. Erkan et al.^17^ compared several programs, including Dolphin, NemoCeph, Quick Ceph^®^, and Vistadent^®^, and reported no significant differences between them. Paixão et al.^18^ also confirmed the reliability of Dolphin software, showing its results were consistent with manual methods. Similarly, Albarakati et al.^19^ found both conventional and digital techniques to be highly reliable, even though they observed small differences in some measurements like ANB angle, convexity angle, and lower anterior facial height (LAFH), which they considered clinically insignificant. Other researchers have highlighted how computer-assisted systems like CADCAS can save time and reduce manual errors.^20^ ^,^ ^21^

Even so, landmark identification remains one of the biggest sources of variability in cephalometric analysis.^22^ ^-^ ^26^ While digital tools help, they still often rely on the operator’s experience and visual judgment, which can be affected by fatigue or other factors. In addition, the high cost of advanced software has sometimes made them less accessible to all clinicians.

More recently, artificial intelligence (AI) has been introduced as a way to improve efficiency and reduce human error in cephalometric analysis. AI-supported software like WebCeph™ and CephNinja are now commonly used.^27^ ^-^ ^29^ These programs automatically locate anatomical landmarks, helping to reduce the orthodontist’s workload and providing easy access to cephalometric analysis through computers or mobile devices. Mahto et al.^30^ showed promising results, reporting good agreement between AI-generated cephalometric measurements from WebCeph™ and traditional manual tracings.

Accurately identifying landmarks is one of the most important steps in cephalometric analysis, because even small errors in positioning can lead to major mistakes in angular and linear measurements. These mistakes can directly affect diagnosis or growth assessment. For example, if landmarks like the porion or nasion are placed incorrectly, it can distort calculations of cranial base length or mandibular plane angles - both of which are crucial for determining skeletal relationships and planning treatment. While many previous studies mainly focused on how accurate the angular or linear measurements were, the present study took a different approach. We specifically examined how much the landmark positions themselves varied by using a coordinate-based comparison across different commercial AI platforms. This provides a clearer picture of where these systems may be inconsistent when marking key anatomical points.

In the present study, we concentrated on the accuracy of landmark placement itself, rather than just measurement outcomes. We used NemoCeph, marked by experienced clinicians, as our gold standard and compared those results to the automatically identified landmarks from WebCeph™ and CephNinja.

Previous research has explored similar comparisons. Kumar et al.^31^ studied Steiner’s analysis using CephNinja and NemoCeph and found no significant differences in skeletal parameters, but minor variations in dental measurements like NA and NB. The present study supports these findings, showing no significant differences at the root tips of upper and lower incisors. In another study, Kumar et al.^32^ found significant differences in Y-axis orientation, the incisor occlusal plane angle, and upper incisor to A-Pog measurements, which aligns with our finding of discrepancies at the porion and incisor apex points.

Duran et al.^33^ compared manual tracing, Dolphin software, OrthoDx™, and WebCeph™, and found acceptable consistency overall, but noted that OrthoDx™ performed slightly better than WebCeph™. In our results, WebCeph™ showed greater consistency than CephNinja, particularly in identifying the porion and incisor apex points.

Çoban et al.^34^ compared Dolphin Imaging and WebCeph™, noting significant differences across several measurements, which they linked to malocclusion type and the difficulty of identifying certain landmarks, like the condyle. Similarly, in the present study, the incisor apex points marked by AI-based software were positioned more distally and superiorly than those identified with NemoCeph, highlighting areas where AI needs further development.

Prince et al.^35^ compared WebCeph™, AutoCEPH^©^, and manual methods, finding strong agreement among them and supporting the use of AI-assisted platforms in clinical practice. The present results are consistent with these findings to a degree. While AI software accurately located most landmarks, we still observed inaccuracies at critical points like porion, basion, gonion, and the incisor apex - points that play an essential role in both angular and linear measurements needed for diagnosis and treatment planning.

Overall, as AI technology advances, it offers great potential to simplify and improve cephalometric analysis. However, clinicians should still carefully review AI-generated landmark placements, especially when dealing with key points that directly influence treatment outcomes. Continued research and algorithm refinement will be necessary to make AI-based cephalometric tools even more reliable and clinically effective.

## LIMITATIONS

A larger sample size would enhance the statistical power and generalizability of the findings.

Blinded evaluations by multiple orthodontists could reduce observer bias and strengthen the validity of the manual tracing results.

Although NemoCeph was considered the reference standard in this study, additional ICC analyses with more raters could help further validate its reliability.

In future studies, the inclusion of additional anatomical landmarks, such as the occlusal plane, may enhance the comprehensiveness of the study.

## CONCLUSION

In this study, we evaluated the accuracy of anatomical landmark identification across manual digital software (NemoCeph) and two AI-based cephalometric software programs (WebCeph™ and CephNinja). It was found that certain anatomical landmarks -porion, basion, gonion, and the apexes of the upper and lower incisors- were located more distally and superiorly in the AI-based software, compared to NemoCeph.

WebCeph™ showed a closer alignment with NemoCeph at the porion and incisor apex points, while CephNinja demonstrated greater similarity at the basion and gonion landmarks. While artificial intelligence has proven effective in most cases, further refinement is required -particularly in points prone to variability or clinical sensitivity.

Clinicians should remain cautious when relying solely on AI-generated cephalometric analyses, and manual validation of key reference points is recommended to ensure diagnostic accuracy and optimal treatment planning.

## Data Availability

Due to sensitive nature of the data and ethical restrictions datasets are not publicly available
